# Phytopathogenic bacterial survival on artificial substrates

**DOI:** 10.17912/micropub.biology.001586

**Published:** 2025-07-11

**Authors:** Emily E. Pfeufer, Aaron J. Sechler, Matthew A. Tancos, Elizabeth E. Rogers

**Affiliations:** 1 Foreign Disease-Weed Science Research Unit, U.S. Department of Agriculture, Agricultural Research Service, Frederick, MD; 2 Current Affiliation: Plant Pest Risk Analysis Unit, U.S Department of Agriculture, Animal and Plant Health Inspection Service, Plant Protection and Quarantine, Raleigh, NC

## Abstract

The ability of phytopathogenic bacteria to survive desiccation on inanimate substrates has important implications for managing potential contamination and resulting bacterial spread during both real-world horticultural operations and laboratory experimentation. Here we demonstrate that *Pseudomonas marginalis, Xanthomonas campestris, Rathayibacter agropyri,* and *R. iranicus* are all capable of surviving desiccation on both polystyrene plastic and glass surfaces and that the likelihood of survival increases with increasing initial bacterial concentration. *X. campestris* was recovered at higher frequencies from plastic than from glass, while the other species were recovered at roughly equal frequencies from each surface.

**Figure 1. Revival numbers and probabilities:**
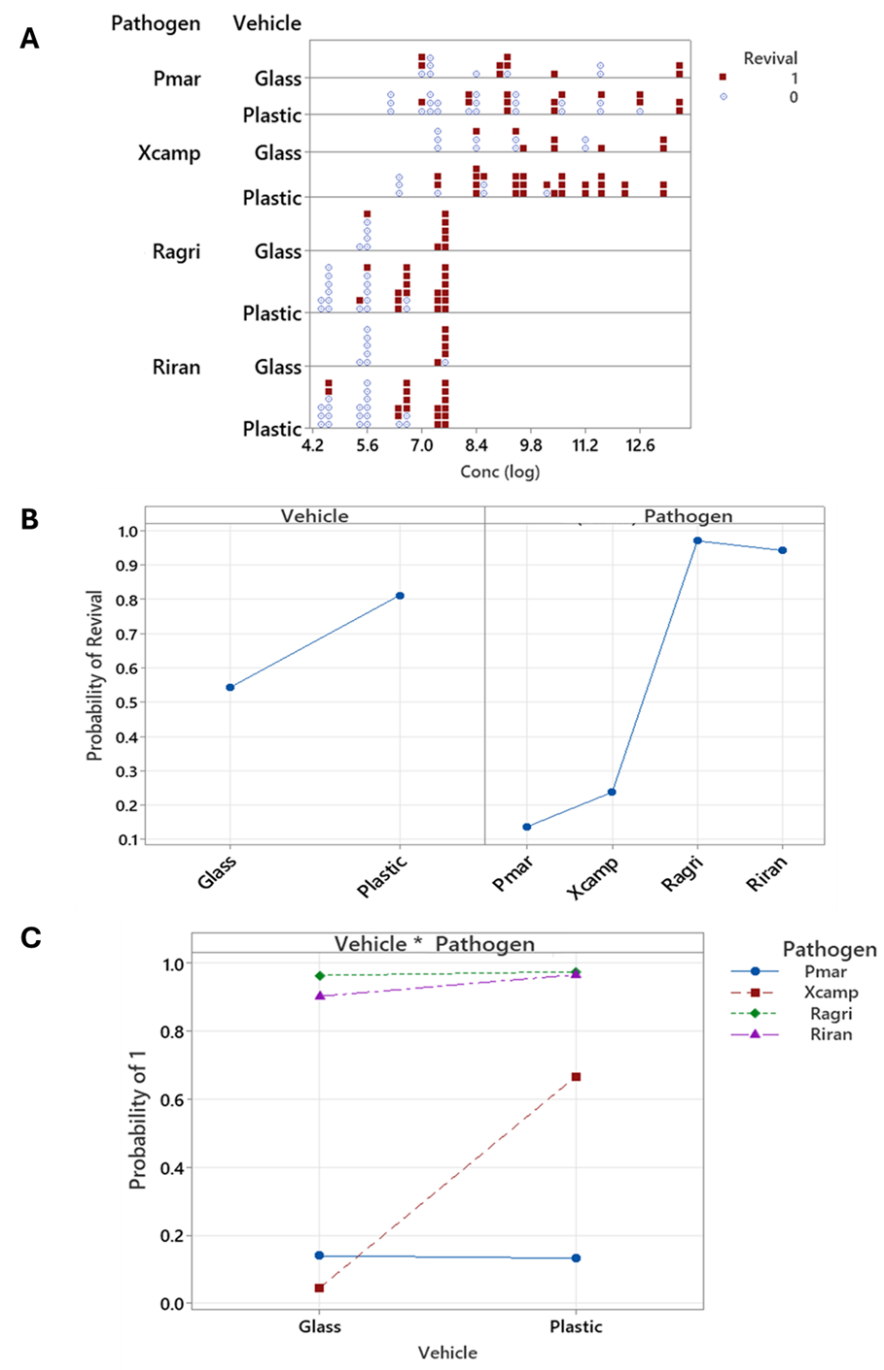
( **A** ) Revival of bacterial strains by species, vehicle, and concentration. Pmarg = *Pseudomonas marginalis* ; Xcamp = *Xanthomonas campestris* ; Ragro = *Rathayibacter agropyri* ; Riran = *Rathayibacter iranicus.* Blue circles = no growth; red square = growth. ( **B** ) main effects plot for revival (denoted by 1) showing vehicle and pathogen; Pmarg = *Pseudomonas marginalis* ; Xcamp = *Xanthomonas campestris* ; Ragro = *Rathayibacter agropyri* ; Riran = *Rathayibacter iranicus.* ( **C** ) interaction plot for revival (denoted by 1) showing Vehicle * Pathogen; blue circles = *P. marginalis* ; maroon squares = *X. campestris* ; green diamonds = *R. agropyri* ; purple triangles = *R. iranicus.*

## Description

Relatively little is known about the ability of phytopathogenic bacteria to survive desiccation on inanimate substrates. Such information is important for managing potential contamination and resulting bacterial spread during both real-world horticultural operations and laboratory experimentation. In the healthcare realm, preventing nosocomial infections is an important contributor to positive patient outcomes, especially when considering immunocompromised patients. Therefore, much effort has been devoted to assessing the persistence of human pathogens on inanimate surfaces (Kramer et al. 2006, Wissmann et al. 2021). In contrast, phytopathogenic bacterial survival on inanimate surfaces has been sparingly addressed in the plant disease literature (Maina and Muthoni 2008, Baysal-Gurel et al. 2015, Alsved et al. 2018, Turechek et al. 2023).

Decontamination procedures for laboratory and greenhouse spaces typically encompass chemical approaches or combinations of heat and pressure to deactivate microbes. Chemical disinfectants include ethanol, sodium hypochlorite (household bleach), and commercially-available greenhouse disinfectants utilizing quaternary ammonium or variations on hydrogen peroxide as active ingredients (Baysal-Gurel et al. 2015, Smith 2015). All chemical disinfectants involve some degree of wetting of materials in order to deactivate microbes, with some options requiring a prolonged soak or pre-washing of all organic matter in order to effectively devitalize microbes (Baysal-Gurel et al. 2015, Smith 2015, Keinath and DuBose 2017). Routine disinfestation of instruments and disposal of experimental materials from microbiology labs typically entails autoclaving, which combines steam and pressure to devitalize materials. Non-autoclavable and non-wettable items, such as electronic equipment and appliances, present a special challenge for decontamination. Bacterial survival following desiccation on commonly used laboratory surfaces, without the application of any disinfectants, will provide baseline survival rates for future devitalization trials using new sanitizing methods for the large-scale disinfection of scientific instruments and appliances.

We examined the ability of four diverse phytopathogenic bacteria to survive desiccation under short room-temperature incubation periods on glass and polystyrene plastic substrates. To incorporate bacterial diversity, two Gram positive and two Gram negative phytopathogenic bacterial species were evaluated: *Rathayibacter agropyri* strain CA-4 isolated from western wheatgrass ( *Pascopyrum smithii* ) (Schroeder et al. 2018), *R. iranicus* strain 66-807 isolated from wheat ( *Triticum aestivum* ) (Zgurskaya et al. 1993), *Xanthomonas campestris* pv. *incanae* strain 18048 isolated from garlic mustard ( *Alliaria petiolata* ) (Tancos et al. 2022), and *Pseudomonas marginalis* strain 22113 isolated from lesser celandine ( *Ficaria verna* ) in Frederick, Maryland (this study).

As shown in Figure 1A, all four bacterial species survived desiccation, although revival (filled red squares) varied based on several factors: strain, starting concentration, and an interaction between strain and substrate (polystyrene plastic or glass; Table 1). Using results from the full statistical model, which included the previous independent variables and interaction terms, the odds of bacterial revival increase 3.9 times (95% confidence interval 2.6 to 5.8) for every additional log of concentration in the starting suspension. Only *X. campestris* showed a revival difference with substrate, with a higher revival probability from polystyrene plastic than from glass ( Figure 1C ). This result differed from recent findings with *Xanthomonas fragariae* -contaminated materials, which suggested no difference in revival from plastic and glass, although only one contaminating concentration (10 ^7^ CFU/ml) was used in that study (Turechek et al. 2023). The present results from *P. marginalis* , with no difference in revival between plastic and glass, combined with those reported from *P. fluorescens* (Bale et al. 1993) and *P. syringae* (Alsved et al. 2018), may suggest survival on different surfaces can vary among species in the same genus.

*R. agropyri* and *R. iranicus,* both Gram positive bacterial strains, had a higher probability of revival than *P. marginalis* and *X. campestris* (which are Gram negative) when other experimental factors were controlled for in the overall statistical model ( Figure 1B ). *Rathayibacter* strains also revived from droplets with lower initial concentrations than *P. marginalis* and *X. campestris* ( Figure 1A ), though further study is warranted to support whether this hypothesis can be broadly applied based on Gram stain of the bacterial plant pathogens. Notably, differences in survival based on Gram stain have been observed with human and animal bacterial pathogens (Bale et al. 1993, Katzenberger et al. 2021, Wissmann et al. 2021), and may not be unexpected given the differences in bacterial cell walls and membranes between the two groups. Importantly, Turechek et al. (2023) demonstrated that not only did *X. fragariae* revive from various surfaces, but it retained its pathogenicity to strawberry plants. While the bacterial strains in the present study were not tested for their subsequent pathogenicity or other changes to their biology after desiccation, continued infectivity to plants would be assumed by their successful revival. More broadly, these results may suggest hypotheses for best working practices with plant pathogenic bacteria when disinfecting sensitive equipment is a serious concern. These would include handling high-titer concentrations with extreme care to prevent spillage and aerosol formation, coupled with regular decontamination of glass and particularly plastic surfaces.

## Methods

To generate bacterial inoculum, a lawn of each species was grown on nutrient rich yeast extract-dextrose-calcium carbonate (YDC) medium (Wilson et al. 1967). The two *Rathayibacter* strains were grown at 28 ^o^ C; *P. marginalis* and *X. campestris* were grown at room temperature (approximately 24 ^o^ C). *P. marginalis* grew in one day, while the other three strains grew in two days. Plates were flooded with sterile distilled water to dislodge the bacteria; liquid was removed and resuspended to an OD _600_ = 0.1. For each replicate experiment, cultures were serially diluted ten-fold and plated to determine starting concentration. Two (trial 1) or four (trials 2, 3, and 4) of the serial dilutions were tested for survival by adding 100 µl bacterial droplets into individual wells in 24-well polystyrene plastic microtiter plates (untreated, manufactured by Fisher or Greiner). Three replicate plates were prepared for each trial. Glass slides were prepared similarly but using only two bacterial concentrations for each species, replicated three times in each trial. Actual CFU range tested was 10 ^6^ – 10 ^13^ for *P. marginalis* and *X. campestris* and 10 ^4^ – 10 ^7^ for *R. agropyri* and *R. iranicus* . Droplets were allowed to dry overnight inside a Class IIA biosafety cabinet with the blower on, then were removed and incubated, uncovered, at room temperature for 10 hours. Droplets were rehydrated with 200 µl of sterile distilled water, mixed by pipetting up and down several times, and 150 µl was spotted onto YDC media to assess viability. Plates were incubated at room temperature (approximately 20 ^o^ C) and scored seven days after replating as a qualitative yes/no. Droplets not resembling the originally infesting strain were discarded as contaminated.

Statistical analysis was completed in Minitab version 21 (Minitab, LLC www.minitab.com ) using n = 197 datapoints which included n = 105 positive revivals. To understand how bacterial strain, concentration, and substrate (plastic or glass) influenced revival, binary data were utilized as the dependent variable in a logistic regression analysis, with 1 = successful revival. “Trial” was included as a class variable to control for variations in concentration among trial dates. Analysis of variance results are displayed with significant variables determined at *P* < 0.05 (Table 1). An odds ratio was used to interpret the effect of initial bacterial concentration on revival in the presence of the other variables in the chosen model.

**Table 1. Analysis of variance.** Effect and significance of bacterial concentration, species, vehicle (glass or plastic), and vehicle*pathogen interaction on revival of four bacterial phytopathogens among four experimental trials. DF = degrees of freedom.

**Table:** 

Source	Wald Test		
	DF	Chi-Square	P-Value
Regression	11	49.66	0.000
Conc (log)	1	45.50	0.000
Substrate/Vehicle	1	00.01	0.939
Species	3	25.46	0.000
Trial	3	19.72	0.000
Vehicle*Species	3	10.48	0.015
